# Hormone-stimulated modulation of endocytic trafficking in osteoclasts

**DOI:** 10.3389/fendo.2012.00103

**Published:** 2012-08-22

**Authors:** Gudrun Stenbeck, Kevin M. Lawrence, Anthony P. Albert

**Affiliations:** ^1^Centre for Cell and Chromosome Biology, School of Health Science and Social Care, Brunel UniversityUxbridge, UK; ^2^Pharmacology and Cell Physiology, Biomedical Sciences Research Centre, Division of Biomedical Sciences, St George’s, University of LondonLondon, UK

**Keywords:** intracellular calcium, calcitonin, endocytosis, osteoclasts, bone resorption

## Abstract

A precise control of vesicular trafficking is crucial not only for osteoclastic bone resorption, but also for the crosstalk between osteoclasts and osteoblasts, which regulates bone homeostasis. In addition to the release of growth factors and modulators, such as glutamate, flux through the intracellular trafficking routes could also provide the osteoclast with a monitoring function of its resorption activity. To establish the signaling pathways regulating trafficking events in resorbing osteoclasts, we used the bone conserving hormone calcitonin, which has the unique property of inducing osteoclast quiescence. Calcitonin acts through the calcitonin receptor and activates multiple signaling pathways. By monitoring trafficking of a fluorescent low molecular weight probe in mature, bone resorbing osteoclasts we show for the first time that calcitonin blocks endocytosis from the ruffled border by phospholipase C (PLC) activation. Furthermore, we identify a requirement for polyunsaturated fatty acids in endocytic trafficking in osteoclasts. Inhibition of PLC prior to calcitonin treatment restores endocytosis to 75% of untreated rates. This effect is independent of protein kinase C activation and can be mimicked by an increase in intracellular calcium. We thus define an essential role for intracellular calcium levels in the maintenance of endocytosis in osteoclasts.

## INTRODUCTION

Bone homeostasis is a dynamic process, which relies on the crosstalk between bone forming osteoblasts, bone resident osteocytes, and bone resorbing osteoclasts (Matsuo, 2012; Xiong and O’Brien, 2012). Imbalances in the crosstalk between these cell types can lead to metabolic bone diseases such as osteoporosis and osteopetrosis (Teti, 2011). This important process is controlled by locally released factors as well as close cell interactions (Cao, 2011). During bone resorption, osteoclasts remove large amounts of extracellular matrix, including growth factors that are stored in the matrix, excavating deep holes in the bone. Even though our understanding of the osteoclast life cycle has increased dramatically over the last decade, the mechanisms that regulate resorption at the cellular level are only starting to emerge (Mellis et al., 2011). At the beginning of the resorption cycle, the plasma membrane in contact with the bone expands through fusion of lysosomes and intracellular vesicles into the highly convoluted ruffled border. The insertion of vacuolar proton ATPase (VATPase) and release of lysosomal proteases provide the microenvironment necessary for resorption of both the organic and inorganic part of the bone (Vaananen and Laitala-Leinonen, 2008). Recent evidence suggests that these exocytic events are controlled by Rab7, synaptotagmin VII, LIS1, and PLEKHM1 (for review see Coxon and Taylor, 2008). Digested material is removed from the resorption pit by endocytosis and trafficked through the osteoclast via a transcytotic route to be released at the functional secretory domain at the bone avert side of the cell (Vaananen and Laitala-Leinonen, 2008). The transcytotic route is important in coupling osteoclast activity with the mesenchymal cell lineage as growth factors such as TGFβ (Karsdal et al., 2007), which are liberated from the bone matrix during resorption, and paracrine signals including glutamate, are released through this route (Morimoto et al., 2006). The extracellular calcium released during the resorption process is also trafficked through the osteoclasts (Yamaki et al., 2005) and plays an important role in mediating osteoclast activity. High extracellular calcium concentrations, as generated in the resorption pit, inhibit osteoclast differentiation and bone resorption (Malgaroli et al., 1989; Mentaverri et al., 2006). However, *in vitro* responses to high extracellular calcium concentrations differ in osteoclasts that are plated on glass to those plated on dentine, indicating that *in vivo* actively resorbing osteoclasts either employ different calcium sensing mechanisms to inactive cells or have very efficient mechanisms of removing extracellular calcium (Lakkakorpi et al., 1996; Stenbeck and Horton, 2000). Capacitive calcium entry and calcium store refilling are important factors in osteoclast survival (Lakkakorpi et al., 1996; Mentaverri et al., 2003). In other cell systems it has been shown that calcium is not only essential for exocytosis but also for intracellular trafficking and endocytosis (Hay, 2007; Pizzo et al., 2011; Shen et al., 2011). Therefore, it is likely that intracellular calcium levels play a role in regulating endocytosis and transcytosis in resorbing osteoclasts.

Most systemic hormones act through osteoblasts to control osteoclast differentiation and activity. However, calcitonin, a peptide hormone secreted by the parafollicular cells of the thyroid and a major player in calcium homeostasis, acts directly on the osteoclast. Calcitonin has rapid inhibitory effects on the activity and morphology of osteoclasts, which include inhibition of trafficking to and from the ruffled border (Lucht, 1973; Baron et al., 1990) and profound alterations of the cytoskeleton (Chambers et al., 1984; Shyu et al., 2007). These effects have a rapid onset and *in vitro* changes to the ruffled border begin to appear after 15 min leading to its almost complete disappearance after 60 min of treatment (Baron et al., 1990). Enzymes located at the ruffled border before calcitonin treatment are found in cytoplasmic vacuoles. The effects of calcitonin are mediated by the calcitonin receptor (CTR), a G protein-coupled receptor linked to Gs, Gi/o, and Gq trimeric G-proteins. Activation of the different G-proteins leads to activation of multiple signaling effectors, including adenylyl cyclase; protein kinase A (PKA); phospholipases C, D, and A2; and protein kinase C (PKC; Horne et al., 1994; Naro et al., 1998; Inzerillo et al., 2002). Recently, the discovery that osteoclasts are having a significant role in providing bone forming signals has led to renewed interest in calcitonin as a treatment for osteoporosis and osteoarthritis (Henriksen et al., 2010). Calcitonin directly targets osteoclasts in a transient manner, which leads to the cessation of resorption without removing the osteoclasts. However, signals leading to the cessation of resorption are poorly defined and it is still unclear what defines pit depth and length. One hypothesis is that flow through the different vesicular trafficking pathways could impart a monitoring function to the osteoclast of its resorption activity. It is thus important to elucidate the mechanisms controlling endocytosis and transcytosis in osteoclasts. During resorption osteoclasts closely attach to the bone matrix with podosomes forming a dense actin ring that encloses the ruffled border. This adhesive structure is restricting access to the resorption area, only allowing access to probes with molecular weights smaller than 10,000 kDa (Stenbeck and Horton, 2000). We have previously shown that low molecular weight markers like TRITC-labeled dextran 3,000 (Stenbeck and Horton, 2004), which have access to the resorption area, are efficiently endocytosed and access the transcytotic route after endocytosis.

Here we use endocytosis of TRITC-labeled dextran 3,000 as an efficient way to establish the signaling pathways regulating trafficking events in mature, resorbing osteoclasts after treatment with the bone conserving hormone calcitonin. To observe uptake of digested material, endocytosis of TRITC-labeled dextran 3,000 is well suited as it follows a similar pathway to the resorbed bone through the osteoclasts and makes labeling of bone and its associated problems, e.g., surface restriction of the label or the necessity of whole animal experiments with tetracycline, redundant. Additionally, with the technique it is possible to analyze immediate early effects elicited by hormones as well as drugs on resorbing cells, thus complementing electrophysiological experiments, which are more difficult to undertake on bone resorbing cells. We show that calcitonin blocks endocytosis from the ruffled border by activating phospholipase C (PLC). Furthermore, we define an essential role for intracellular calcium oscillations in the maintenance of endocytosis and transcytosis during resorption.

## MATERIALS AND METHODS

### MATERIALS

 Fluorescent dextran (TRITC-dextran, MW of 3,000), Alexa-Fluor488- and AlexaFluor647-conjugated phalloidin as well as AlexaFluor488-labeled goat anti-mouse secondary antibodies were from Molecular Probes (Leiden, Netherlands). Tissue culture materials were obtained from Invitrogen (Paisley, UK). Salmon calcitonin, the PLC inhibitor 1-[6-[[(17β)-3-methoxyestra-1,3,5(10)-trien-17-yl]amino]hexyl]-1 *H*-pyrrole-2,5-dione (U-73122) and its inactive analog U-73343; protein kinase A inhibitor *N*-[2-[[3-(4-bromophenyl)-2-propenyl]amino]ethyl]-5-isoquinolinesulfonamide dihydrochloride (H-89), cell-permeable calcium chelator BAPTA-AM, ionomycin, thapsigargin, protein kinase C inhibitor bisindolylmaleimide I (BIS) and chelerythrine chloride, protein kinase C activator phorbol-12-myristate-13-acetate (PMA), phosphodiesterase inhibitor 3-isobutyl-1-methylxanthine (IBMX), and 8-bromo-cAMP were from Calbiochem (Nottingham, UK). cAMP-dependent protein kinase inhibitor Rp-cAMPS was from Biomol (Enzo Life Sciences, Exceter, UK). 6-monobutyric cAMP (6-MB-cAMP), diacylglycerol kinase inhibitor 3-[2-[4-[*bis*(4-Fluorophenyl)methylene]-1-piperidinyl] ethyl]-2, 3-dihydro-2-thioxo-4(1*H*)-quinazolinone (R59949) and diacylglycerol lipase inhibitor *O*,*O*’-[1,6-Hexanediyl *bis*(iminocarbonyl)]dioximecyclohexanone (RHC 80267) were from Sigma (Poole, UK). Stock solutions of the inhibitors were prepared in DMSO and in every relevant experiment an equivalent volume of DMSO was added to control samples. Anti-α_v_β_3_ antibodies (clone 23C6) were described previously (Davies et al., 1989).

### ISOLATION AND CULTURE OF OSTEOCLASTS

 To assess the modulation of trafficking during bone resorption we used osteoclasts derived from neonatal rabbits as a readily available source of mature osteoclasts with morphology similar to human osteoclasts. The procedure used to culture rabbit osteoclasts was modified from the original method developed by Tezuka et al. (1992) and as described in Stenbeck and Horton (2004). Briefly, osteoclasts were mechanically disaggregated from long bones of 5-day old rabbits in α-MEM containing 2 mM L-glutamine, 100 i.u./ml penicillin, 100 μg/ml streptomycin. The cells were then pelleted, resuspended in α-MEM containing 2 mM L-glutamine, 100 i.u./ml penicillin, 100 μg/ml streptomycin, 10% fetal bovine serum (FBS) and allowed to attach to sonicated dentine slices (100–150 μm thick, surface area 0.3 cm^2^) at 37°C in 5% CO_2_/95% air for 60 min. Non-adherent cells were then washed away and the remaining cells were cultured for 20 h in α-MEM containing 2 mM L-glutamine, 100 i.u./ml penicillin, 100 μg/ml streptomycin, and 10% FBS.

### MEASUREMENT OF ENDOCYTOSIS

 To measure endocytosis from the ruffled border, osteoclasts on dentine slices were incubated overnight and then treated with fluorescent-labeled dextran (TRITC-dextran) as described in Stenbeck and Horton (2004). Briefly, cells on dentine slices were transferred into α-MEM buffered with 20 mM HEPES (pH 7.0) containing 2 mM L-glutamine, 100 i.u./ml penicillin, 100 μg/ml streptomycin, and 0.1% BSA (MEM-BSA) and incubated for 5 min in MEM-BSA containing 260 μM of TRITC-labeled dextran 3,000. Routinely between 48 and 75 % of integrin α_v_β_3_ positive multinucleated cells were endocytosis positive. The incubations were stopped by washing the cells on dentine slices twice with PBS for 20 s followed by the immediate addition of 3% paraformaldehyde, 2% sucrose in PBS for 10 min at RT. Images shown are representative of a minimum of 50 different osteoclasts.

 For pharmacological experiments, osteoclasts on dentine slices were pre-incubated for 5–30 min at 37°C in MEM-BSA containing the appropriate drug before addition of TRITC-labeled dextran 3,000. Inhibitor concentrations were chosen according to their ability to inhibit osteoclast resorption in overnight pit forming assays or their effects in electrophysiology experiments (Bennett et al., 2001; Albert et al., 2005) or as reported in the literature (Murrills and Dempster, 1990; Lakkakorpi et al., 1996; Chen et al., 2002). In each experiment three independent dentine slices were used and experiments were repeated at least twice with cells from different rabbits. The total number of osteoclasts that were analyzed per inhibitor treatment varied between 350 and 670.

### IMMUNODETECTION

 Resorbing osteoclasts were identified by immunostaining with anti-α_v_β_3_ antibodies (clone 23C6) and/or staining with fluorophore-conjugated phalloidin. After fixation the cells were incubated for 30 min in 5% newborn bovine serum in PBS containing 0.02% sodium azide (wash) and then processed for immunodetection. Primary antibodies were diluted in wash and the cells were incubated for 30 min at RT. Bound antibodies were detected with AlexaFluor 488-conjugated goat anti-mouse secondary antibodies. For triple-labeling experiments cells stained with anti-α_v_β_3_ antibodies were incubated with AlexaFluor 647-conjugated phalloidin for 30min at RT.

### MICROSCOPY

 TRITC-labeled dextran and antibody/actin distribution was monitored with a Leica TCS-NT confocal laser-scanning microscope (CSLM; Heidelberg, Germany), using standard filter settings and sequential scanning to avoid overlap of emission from the fluorophores. The thickness of the optical section was set to 1.013 μm for the *xy* sections and to 1.008 μm for the *zx* sections. In some images the *xy* sections were added together with the extended focus option of the Leica TCS-NT software to allow the visualization of the area just above the bone surface to the bottom of the pit. The *xy* sections were used to count the number of endocytosis-positive osteoclasts. Osteoclasts were considered endocytosis-positive if they showed robust red vesicular staining in the resorption area; *zx* sections were used to confirm endocytosis if *xy* sections were ambiguous.

### STATISTICAL ANALYSIS

 All experiments were repeated at least twice and in each experiment the number of α_v_β_3_ antibody-positive, multinucleated cells was determined and examined for TRITC-labeled dextran 3,000 endocytosis. In experiments with inhibitors, at least 350 osteoclasts were observed to determine the number of endocytosis-positive cells. Results obtained for each experiment were expressed as percentage of endocytosis-active osteoclasts over total number of osteoclasts (±SD) and compared to vehicle-treated controls. In some experiments the number of endocytosis-active osteoclasts was set to zero to visualize the effect of the treatment on restoring control conditions, i.e., inhibiting the effect of calcitonin. Results from two to five independent experiments were pooled before plotting. We determined the normal distribution of the data by fitting a Gaussian function on the entire dataset. Differences were analyzed by Student’s *t*-test and considered significant if *p* < 0.05.

## RESULTS

### CALCITONIN AND CAMP ELEVATION INTERFERE WITH ENDOCYTOSIS IN ACTIVELY RESORBING OSTEOCLASTS

**FIGURE 1:**
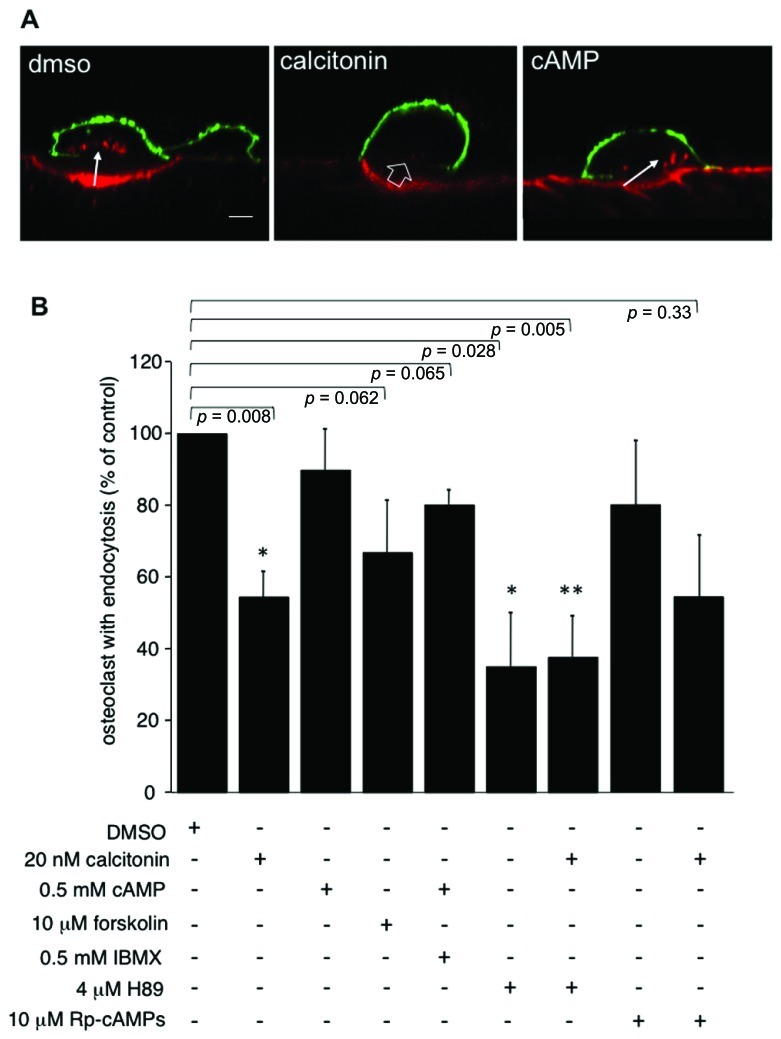
** Incubation of osteoclasts with 20 nM calcitonin for 30 min reduces endocytosis in osteoclasts.** This effect is only partially mimicked by addition of forskolin to directly activate adenylate cyclase, or by raising intracellular cAMP with 6-MB-cAMP (cAMP) alone or together with the phosphodiesterase inhibitor IBMX. Inhibiting signaling pathways downstream of cAMP does not reverse the calcitonin effect. Exposure of osteoclasts to 4 μM of the PKA inhibitor H89 for 30 min lowers the number of endocytosis-active osteoclasts to a level comparable to calcitonin addition. Incubation of the cells with H89 for 5 min before the addition of calcitonin does not reverse the calcitonin-mediated inhibition of endocytosis. The inhibitory analog of cAMP, Rp-cAMPS, has only limited effects on endocytosis on its own and does not reverse the response to calcitonin. **(A)** Lateral view of rabbit osteoclasts plated on dentine after incubation with vehicle, calcitonin or 6-MB-cAMP for 30 min. Red, TRITC-dextran; arrow, endocytosed TRITC-dextran; open arrow, ruffled border area without endocytosis; green, anti-α_v_β_3_ antibodies. Scale bar: 10 μm. **(B)** Chart showing the inhibitory effect of the different drugs when compared to endocytosis in vehicle-treated cells. Results are average of three independent experiments. Error bars represent standard deviation, significant reductions in dextran endocytosis compared to control are marked: *p < 0.05, **p < 0.005, individual *p*-values are shown above the bars.

In accordance with previously published results (Lucht, 1973; Baron et al., 1990), pre-incubation of rabbit osteoclasts plated on dentine slices with 20 nM calcitonin for 30 min markedly reduces the number of osteoclasts showing endocytosis compared to control samples (**Figures 1A,B**). In other cell systems cAMP and subsequent activation of PKA has been shown to interfere with endocytosis and transcytosis (Bradbury et al., 1992; Hansen and Casanova, 1994; Cotlin et al., 1999). We show here that in osteoclasts elevating the intracellular cAMP concentration by adding a membrane permeable cAMP analog (Kopperud et al., 2003) and the phosphodiesterase inhibitor IBMX has only a limited effect on osteoclast endocytosis from the ruffled border (**Figures 1A,B**). In our experiments, we used both 8-bromo-cAMP, which also activates exchange-protein-directly activated-by-cAMP (Epac), and monobutyl cAMP (6-MB-cAMP), which is specific for PKA (Cheng et al., 2008). Using these two cAMP analogs we obtained identical results (11% ± 11.2 reduction in the number of osteoclast with endocytosis). However, when adenylate cyclase was directly activated by forskolin, the number of osteoclasts with endocytosis was reduced below the level obtained with these cAMP analogs alone or together with the phophodiesterase inhibitor IBMX (67% ± 14; 89% ± 11.2; 80% ± 4, respectively; **Figure 1B**). These results indicate that cAMP signaling effectors other than PKA may play a role in regulating endocytosis in osteoclasts. We thus tested the effect of inhibiting PKA directly with either H89 or Rp-cAMPS. These inhibitors have different modes of action; H89 blocks the ATP binding site of PKA whereas Rp-cAMPS is an inhibitory analog of cAMP (Lochner and Moolman, 2006). After overnight culture, osteoclasts on dentine were pre-incubated for 5 min with H89 or for 10 min with Rp-cAMPS, before addition of calcitonin for 30 min and subsequent addition of TRITC-labeled dextran 3,000 for 5 min. Treatment with 4 μM H89 for 35 min on its own statistically significantly lowered the number of endocytosis-active osteoclasts by 64% to 36% ± 14.8, and the addition of calcitonin had no further effect. In contrast, we observed only a mild inhibition with Rp-cAMPS (16.9% ± 18.3) and a response to calcitonin similar to vehicle-treated cells (54.3% ± 18.1; **Figure 1B**). These findings clearly indicate that as observed in other cell systems, H89 inhibits a number of different pathways that are distinct from PKA (Lochner and Moolman, 2006; Palacios et al., 2007).

### PLC SIGNALING MEDIATES THE INHIBITORY EFFECT OF CALCITONIN ON ENDOCYTIC TRAFFICKING

**FIGURE 2:**
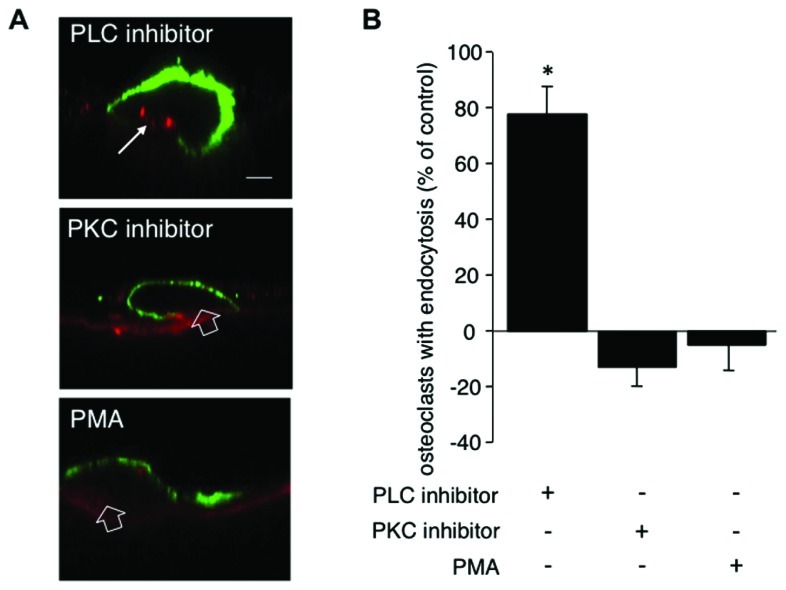
** PLC but not PKC signaling conveys the inhibitory effect of calcitonin on endocytosis.** Osteoclasts plated on dentine for 16 h were pre-incubated for 5 min with either the PLC inhibitor U-73122 (1 μM) or the PKC inhibitor bisindolylmaleimide (1 μM) or the PKC activator PMA (1 μM) before the addition of calcitonin for 30 min. TRITC-dextran 3,000 was then added for 5 min before fixation. Cells were immunodecorated with antibodies directed against α_v_β_3_ integrin. **(A)** Representative lateral view of treated cells. Red, TRITC-dextran; arrow, endocytosed TRITC-dextran; open arrow, ruffled border area without endocytosis; green, anti-α_v_β_3_ antibodies. Scale bar: 10 μm. Treatment with the PLC inhibitor restores endocytic trafficking whereas neither the PKC inhibitor nor the PKC activator PMA has an effect. **(B)** The number of osteoclasts displaying endocytosed dextran was counted and expressed as percentage of total. Columns show mean percentage of osteoclasts with dextran endocytosis compared to calcitonin treatment alone (set to 0). Results are average of three independent experiments. Error bars show standard deviation and significant increases in endocytosis-active osteoclasts are marked: *p < 0.05. Treatment with the PLC inhibitor restores endocytic trafficking to 77% whereas neither inhibiting nor activating PKC has an effect on the reduction in endocytosis-active osteoclasts after calcitonin treatment.

To investigate whether the calcitonin-mediated inhibition of endocytic trafficking requires the PLC pathway, after overnight culture osteoclasts on dentine slices were incubated for 5 min with 1 μM of the PLC inhibitor U-73122 before addition of calcitonin. Treatment with the inhibitor restores endocytic trafficking to 77% of control cells (**Figures 2A,B**). PLC signaling gives rise to two second messengers, DAG and IP_3_. IP_3_ activates calcium efflux from intracellular stores whereas DAG, in conjunction with calcium, activates PKC and several other molecules, as recently reported (Sossin and Farah, 2009).

To investigate the role of PKC in osteoclast endocytosis, we pre-treated the cells with the PKC inhibitors bisindolylmaleimide or chelerythrine for 5 min before the addition of calcitonin. Treatment with both inhibitors resulted in reduction of endocytosis to a level below that observed with calcitonin alone (43% ± 6.6 versus 54.5% ± 7.9, respectively; **Figure 2B**). In contrast to the results obtained with H89, pre-treatment with the PKC inhibitors had no significant effect on endocytic trafficking (92% osteoclasts showed endocytosis compared to control; data not shown). To test whether PKC activation independent of PLC activation can interfere with calcitonin signaling to the endocytic machinery in osteoclasts, we treated the osteoclasts for 5 min with Phorbol-12-Myristate-13-acetate (PMA), which directly activates PKC. Pre-treatment with PMA did not reverse calcitonin-induced reduction in the number of endocytosis-active osteoclasts (50.95% ± 12.09; **Figure 2B**).

### POLYUNSATURATED FATTY ACIDS BUT NOT DAG ARE IMPORTANT FOR ENDOCYTIC TRAFFICKING

**FIGURE 3:**
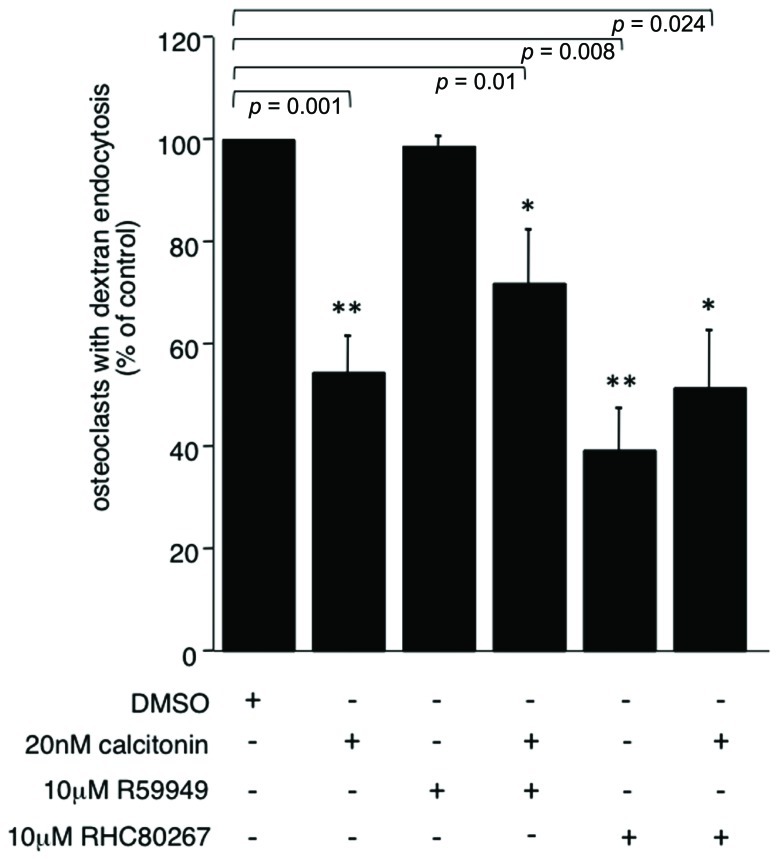
** PUFA are important for endocytic trafficking.** Rabbit osteoclasts plated on dentine were incubated for 16 h before a 15 min pre-incubation with either the DAG kinase inhibitor R55949 or the DAG lipase inhibitor RHC 80267 (both 10 μM) before the addition of 20 nM calcitonin for 30 min. TRITC-dextran 3,000 was then added for 5 min before fixation. The number of osteoclasts presenting endocytosed dextran was counted after immunodecoration with antibodies directed against the α_v_β_3_ integrin and expressed as percentage of total. Columns show mean percentage of osteoclasts with dextran endocytosis and standard deviation. Results are average of two independent experiments. Significant reductions in dextran endocytosis are marked: *p < 0.05, **p < 0.005, individual *p*-values are shown above the bars.

 To further elucidate the pathway by which PLC activation inhibits endocytic trafficking in osteoclasts we used the DAG kinase inhibitor R59949 and the DAG lipase inhibitor RHC 80267 to increase the intracellular DAG levels. DAG kinases are the main enzymes responsible for termination of the DAG signal. In recent years, it has become clear that DAG can activate targets other than PKC, some of which are important for vesicle fusion. A direct activity onion channels has also been reported (Sossin and Farah, 2009). Interestingly, increasing the concentration of DAG with the DAG kinase inhibitor did not reverse the calcitonin effect (66.3% ± 10.3 versus 54.5% ± 7.9, respectively; **Figure 3**). However, inhibiting the production of polyunsaturated fatty acids (PUFA) by blocking DAG lipase had a statistically significant negative effect on endocytic trafficking (39% ± 8.06; **Figure 3**) and did not reverse the calcitonin effect, highlighting the importance of fatty acids in this process (Stock and Coderre, 1984; Brown et al., 2003; Spilsberg et al., 2007).

### CALCIUM DYNAMICS ARE NECESSARY FOR ENDOCYTIC TRAFFICKING AND ARE AFFECTED BY CALCITONIN TREATMENT

**FIGURE 4:**
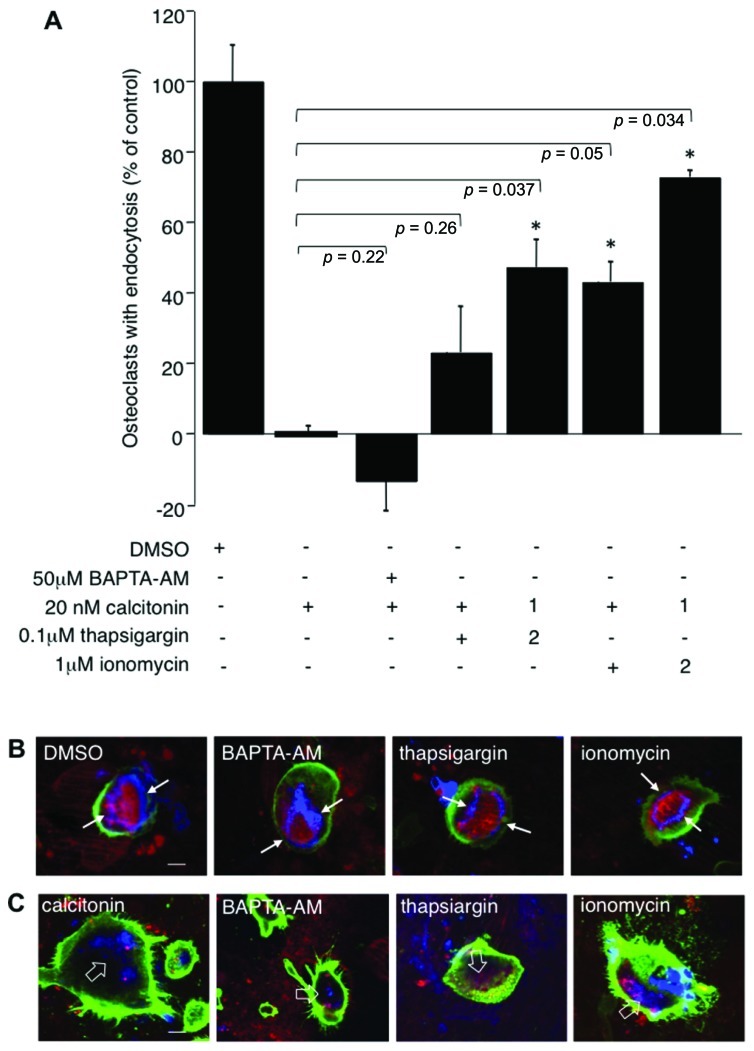
** Calcium fluctuations are essential for endocytic trafficking from the ruffled border.** Rabbit osteoclasts plated on dentine for 16 h were incubated with 20 nM calcitonin and indicated concentrations of calcium modulators for 30 min before the addition of TRITC-dextran 3,000 for 5 min and fixation. 1 μM ionomycin or 100 nM thapsigargin were either added together with calcitonin (1) or were added 1.5 min after addition of calcitonin (2). **(A)** Chart showing the effects of different inhibitors. The number of osteoclasts presenting endocytosed dextran was counted after immunodecoration with antibodies directed against the α_v_β_3_ integrin and expressed as percentage of total. Columns show mean percentage of osteoclasts with dextran endocytosis in comparison to calcitonin treatment alone (set to 0). Error bars represent SD, significant increases in dextran endocytosis over calcitonin treatment are marked: *p < 0.05, individual *p*-values are shown above the bars. **(B)** Morphology of the actin-cytoskeleton after exposure to calcium modulators. Rabbit osteoclasts plated on dentine were exposed for 30 min to 50 μM BAPTA-AM; 1 μM ionomycin; 100 nM thapsigargin before addition of TRITC-dextran 3,000 for 5 min, fixation and immunodecoration. Red, TRITC-dextran; green, anti-α_v_β_3_ antibodies; blue, actin cytoskeleton. The resorption area enclosed by the actin ring is indicated with arrows. **(C)** Osteoclasts on dentine were incubated for 30 min with 20 nM calcitonin together with 50 μM BAPTA-AM; 1 μM ionomycin or 100 nM thapsigargin. Red, TRITC-dextran; green, anti-α_v_β_3_ antibodies, blue, actin-cytoskeleton. Disorganized actin filaments in the cells treated with calcitonin are highlighted with open arrows. Scale bars: 10 μm.

PLC activation increases intracellular calcium via the generation of IP_3_ and calcium release from internal stores. To study the influence of calcium on endocytic trafficking after calcitonin treatment, we used several independent approaches. First, we clamped the intracellular calcium concentration via BAPTA-AM, a cell permeable calcium chelator. Treatment of cells with calcitonin in the presence of 50 μM BAPTA-AM augmented the inhibitory effect of calcitonin (59.7% ± 1.8 compared to 46.8% ± 7.9 with calcitonin alone), indicating that intracellular calcium plays an important role in the regulation of endocytosis (**Figure 4A**). Treatment of osteoclasts with BAPTA-AM alone for 30 min had only a modest inhibitory effect on the number of endocytosis-positive osteoclasts (22% ± 9.2; data not shown).

To check involvement of calcium release from intracellular stores we used the ionophore ionomycin and thapsigargin, which interferes with refilling of intracellular stores by inhibiting sarcoplasmic/endoplasmic reticulum Ca^2+^-dependent ATPase pump (SERCA). Interestingly, both ionomycin and thapsigargin restored endocytic activity in osteoclasts after calcitonin treatment and had their largest effect when added soon after addition of calcitonin. Incubation of calcitonin together with thapsigargin or ionomycin restored endocytic trafficking to 23 (±13.05) and 43% (±5.75), respectively. However, addition of ionomycin or thapsigargin 1.5 min after calcitonin restored trafficking to 47 (±7.8) and 72.7% (±1.9) of control, respectively (**Figure 4A**). In agreement with previously published results, the actin cytoskeleton was not affected by treatment with the intracellular calcium modulators (BAPTA-AM, thapsigargin or ionomycin) for 30 min (Lakkakorpi et al., 1996; **Figure 4B**). In contrast to the reversal of the inhibitory effect of calcitonin on intracellular trafficking, treatment with the intracellular calcium modulators did not overturn the effect that calcitonin has on the actin cytoskeleton (**Figure 4C**). Taken together with the results obtained using the PKC inhibitors these findings strongly suggest that calcitonin affects endocytic trafficking by reducing intracellular calcium levels in osteoclasts.

## DISCUSSION

A precise control of vesicular trafficking is crucial for endocytosis and exocytosis in osteoclasts, and as such, for bone homeostasis. Factors regulating membrane trafficking in these cells are therefore paramount for controlling bone dynamics and may represent novel avenues for therapeutic intervention in bone pathologies. The recent discovery that osteoclasts play a significant role in providing bone-forming signals has led to renewed interest in calcitonin as a treatment for osteoporosis and osteoarthritis (Henriksen et al., 2010). Here we used calcitonin as a tool to investigate the regulation of endocytic trafficking in mature osteoclasts. Calcitonin elicits multiple effects on osteoclasts, which include cessation of intracellular trafficking to and from the ruffled border (Lucht, 1973; Baron et al., 1990) via a complex signaling network (Inzerillo et al., 2002).

 Independent evidence demonstrates the effect of calcitonin on the osteoclast actin cytoskeleton, which leads to a decrease in cell motility. These effects are mediated by high intracellular cAMP. So far, no experimental evidence links cAMP signaling events to the cessation of membrane trafficking after calcitonin treatment. In other cell types, cAMP has been shown to stimulate exocytosis and to reduce clathrin-mediated and fluid phase endocytosis (Bradbury et al., 1992; Gekle et al., 1995; Bouley et al., 2011) as well as to enhance transcytosis (Hansen and Casanova, 1994). We were thus interested to determine the effect of elevating intracellular cAMP levels on TRITC-labeled dextran 3,000 endocytosis. We increased intracellular cAMP by using membrane permeable cAMP analogs, forskolin or via PKA activation. PKA is a well-known target of cAMP but there are several other molecules, most notably the Epacs, that are stimulated by a rise in intracellular cAMP concentration (Cheng et al., 2008). Our results show that forskolin has a more pronounced effect on the number of endocytosis-active osteoclasts than the cell-membrane permeable cAMP analogs, suggesting that the calcitonin effect on endocytosis is partially due to a combination of Epac- and PKA-derived downstream signals. However, the fact that the cAMP analog 8-bromo-cAMP, which activates both PKA and Epac, did not produce different results to the PKA-specific 6-MB-cAMP indicates that there might be other cAMP-sensitive signaling pathways triggered by calcitonin that are mediating its inhibitory effects on endocytosis. This hypothesis is supported by the results obtained with the established PKA inhibitors H89 and Rp-cAMPS, which failed to restore endocytosis after calcitonin treatment. Instead, we found that H89 profoundly reduced the number of endocytosis-active osteoclasts both with and without calcitonin treatment. Even though a negative effect of PKA inhibition on vesicular trafficking has been demonstrated in other cell systems (Butterworth et al., 2005; Bejarano et al., 2006; Wojtal et al., 2008), there is also evidence that H89 inhibits several other kinases at concentrations similar to those blocking PKA (Lochner and Moolman, 2006). This is also the most likely scenario in our experiments, since the inhibitory cAMP analog (Rp-cAMPS) did not show the same effects as H89. Taken together, our results demonstrate that cAMP-dependent signaling is not essential in the inhibition elicited by calcitonin on the endocytic machinery, and that the effect observed with H89 in osteoclasts is due to the inhibition of another kinase (Lee and Linstedt, 2000; Lochner and Moolman, 2006).

The other classical signaling pathway activated by calcitonin is the PLC- PKC-calcium pathway (Su et al., 1992). To establish whether this pathway is involved in the calcitonin-dependent modulation of the endocytic machinery, we used a PLC inhibitor, a PKC inhibitor as well as a PKC activator. PKC activation has been shown to induce changes in tyrosine phosphorylation of cell adhesion kinases, which leads to disruption of the peripheral actin ring in osteoclasts and is involved in cell retraction after calcitonin treatment (Zhang et al., 2002; Shyu et al., 2007). Interestingly, the PLC inhibitor reversed the calcitonin effect and restored endocytic trafficking to almost control levels. Surprisingly, however this effect was not mediated by PKC. In recent years, it has become clear that there are other factors besides PKC that are stimulated by second messengers generated by PLC activation, such as DAG. DAG on its own has a number of membrane traffic relevant effects, including regulation of SNARE complex formation and direct ion channel stimulation (Sossin and Farah, 2009). Additionally, products of DAG inactivation by DAG lipases (PUFA) and kinases (phosphatidic acid) have been shown to modulate intracellular trafficking (Darios et al., 2007; Donaldson, 2009; Davletov and Montecucco, 2010; Malhotra and Campelo, 2011). We thus investigated the effect of increasing DAG by using the specific DAG lipase inhibitor RHC 80267 and DAG kinase inhibitor R59949. Elevated DAG levels had no effect on calcitonin-mediated inhibition of TRITC-dextran 3,000 endocytosis, but blocking the generation of PUFA with RHC 80267 decreased the number of endocytosis-active osteoclasts. These results clearly demonstrate an important role of PUFA in osteoclasts activity, in addition to their role in osteoclast differentiation (Kruger et al., 2010; Yuan et al., 2010).

 PLC activation increases intracellular calcium, an effect also observed upon calcitonin treatment (Malgaroli et al., 1989; Xia and Ferrier, 1996). When the concentration of intracellular calcium was increased using thapsigargin, a SERCA inhibitor, or the ionophore ionomycin, endocytic trafficking was restored in calcitonin-treated cells. This recovery was almost complete when the calcium increase followed a brief incubation with calcitonin. These results are similar to what has been previously observed by Malgaroli et al. (1989) where a brief pre-incubation with calcitonin augmented the rise in intracellular calcium elicited by high extracellular calcium levels. Xia and Ferrier (1996) showed that calcitonin stimulated localized calcium changes, which consisted of repetitive short-lasting spikes and single pulses in different areas of the cells. It will be thus important in the future to measure intracellular calcium changes in response to inhibitor and calcitonin treatment in situ by loading resorbing osteoclasts on bone with fluorescent calcium indicators such as Fura-2 AM or Fluo-4 AM. These experiments will also further our studies on the effects of calcitonin on transient receptor potential (TRP) channels (Albert et al., 2010). TRP channels have recently received much interest because they have been found to regulate osteoclast differentiation and calcium sensing (Bennett et al., 2001; van der Eerden et al., 2005; Guilak et al., 2010). TRP channels have a wide range of functions, one of which is store-operated calcium entry after depletion of intracellular stores. It is thus possible that the initial rise in intracellular calcium concentration after treatment of the cells with thapsigargin or ionomycin is sustained by influx of calcium through store-operated channels. Since sustained increases in intracellular calcium trigger osteoclast apoptosis (Mentaverri et al., 2003), this effect should be short lived in order for cells to survive. As calcitonin does not cause osteoclast apoptosis, it is unlikely that a sustained intracellular calcium elevation is taking place after calcitonin treatment. This hypothesis is supported by our findings on the morphology of the actin cytoskeleton after treatment with calcium modulators. Previous reports have shown a profound effect of increased intracellular calcium on osteoclast cytoskeleton. In contrast, we did not observe actin redistribution after incubating the cells for 30 min with calcium modulators. These results are in agreement to those reported by Lakkakorpi et al. (1996) demonstrating a very short effect of ionomycin on the actin cytoskeleton in osteoclasts plated on bone slices.

 Taken together, our results clearly suggest an important role for calcium in osteoclast endocytosis. Recently, thapsigargin has been shown to inhibit autophagic flow by blocking the fusion of autophagosomes with lysosmes (Ganley et al., 2011). As autophagy genes, such as Atg5, play an important role in osteoclast ruffled border formation we cannot rule out a compensatory effect on endocytic trafficking induced by thapsigargin. However Atg5 which is also involved in thapsigargin-induced autophagy, does not play a role in endosome uptake and trafficking from the ruffled border (DeSelm et al., 2011). It is thus more likely that it is the thapsigargin-induced calcium release that is relieving the calcitonin-induced block in endocytic trafficking. In neurons and chromaffin cells, a rise in intracellular calcium triggers exocytosis, however, other trafficking steps, such as endocytosis also depend on intracellular calcium (Hosoi et al., 2009; Rosa et al., 2011). These findings are in line with our observations that clamping intracellular calcium with the cell-permeable calcium chelator BAPTA-AM reduced the number of endocytosis-active osteoclasts and did not reverse the effect of calcitonin.

 In summary, we have identified an important regulatory function of calcium signaling in endocytosis from the ruffled border in osteoclasts. Increasing intracellular calcium may modulate the endocytic machinery through calcineurin, calmodulin, and/or calmodulin-dependent protein kinase II regulation of components of membrane traffic machinery, such as dynamin (Smillie and Cousin, 2005), EEA1, and syntaxin (Risinger and Bennett, 1999) in addition to facilitating bilayer fusion (Peters and Mayer, 1998). Synaptotagmins are calcium sensors involved in membrane fusion and synaptotagmin VII has been shown to be essential for the regulation of exocytosis towards the ruffled border in osteoclasts (Zhao et al., 2008). In agreement with the recently described role of synaptotagmin I in clathrin-mediated endocytosis at nerve terminals (Yamashita, 2012), our findings suggest the exciting possibility that an increase in intracellular calcium couples exo- and endocytosis in osteoclasts via synaptotagmin VII.

## Conflict of Interest Statement

The authors declare that the research was conducted in the absence of any commercial or financial relationships that could be construed as a potential conflict of interest.

## References

[B1] Albert A. Chambers T. Lawrence K. (2010). Calcitonin and alendronate activate Ca^2+^-permeable cation conductances with distinct transient receptor potential properties in murine osteoclasts. *Bone* 46 S60

[B2] Albert A. P. Piper A. S. Large W. A. (2005). Role of phospholipase D and diacylglycerol in activating constitutive TRPC-like cation channels in rabbit ear artery myocytes. *J. Physiol.* 566 769–780 15919706 10.1113/jphysiol.2005.090852 PMC1464787

[B3] Baron R. Neff L. Brown W. Louvard D. Courtoy P. J. (1990). Selective internalization of the apical plasma membrane and rapid redistribution of lysosomal enzymes and mannose 6-phosphate receptors during osteoclast inactivation by calcitonin. *J. Cell Sci.* 97 439–447 1963625 10.1242/jcs.97.3.439

[B4] Bejarano E. Cabrera M. Vega L. Hidalgo J. Velasco A. (2006). Golgi structural stability and biogenesis depend on associated PKA activity. *J. Cell Sci.* 119 3764–3775 16926194 10.1242/jcs.03146

[B5] Bennett B. D. Alvarez U. Hruska K. A. (2001). Receptor-operated osteoclast calcium sensing. *Endocrinology* 142 1968–1974 11316762 10.1210/endo.142.5.8125

[B6] Bouley R. Lu H. A. Nunes P. Da Silva N. Mclaughlin M. Chen Y. Brown D. (2011). Calcitonin has a vasopressin-like effect on aquaporin-2 trafficking and urinary concentration. *J. Am. Soc. Nephrol.* 22 59–72 21071524 10.1681/ASN.2009121267 PMC3014035

[B7] Bradbury N. A. Jilling T. Kirk K. L. Bridges R. J. (1992). Regulated endocytosis in a chloride secretory epithelial cell line. *Am. J. Physiol.* 262 C752–C759 1312784 10.1152/ajpcell.1992.262.3.C752

[B8] Brown W. J. Chambers K. Doody A. (2003). Phospholipase A2 (PLA2) enzymes in membrane trafficking: mediators of membrane shape and function. *Traffic* 4 214–221 12694560 10.1034/j.1600-0854.2003.00078.x

[B9] Butterworth M. B. Frizzell R. A. Johnson J. P. Peters K. W. Edinger R. S. (2005). PKA-dependent ENaC trafficking requires the SNARE-binding protein complexin. *Am. J. Physiol. Renal Physiol.* 289 F969–F977 15972388 10.1152/ajprenal.00390.2003

[B10] Cao X. (2011). Targeting osteoclast-osteoblast communication. *Nat. Med.* 17 1344–1346 22064408 10.1038/nm.2499

[B11] Chambers T. J. Athanasou N. A. Fuller K. (1984). Effect of parathyroid hormone and calcitonin on the cytoplasmic spreading of isolated osteoclasts. *J. Endocrinol.* 102 281–286 6481285 10.1677/joe.0.1020281

[B12] Chen J. L. Ahluwalia J. P. Stamnes M. (2002). Selective effects of calcium chelators on anterograde and retrograde protein transport in the cell. *J. Biol. Chem.* 277 35682–35687 12114519 10.1074/jbc.M204157200

[B13] Cheng X. Ji Z. Tsalkova T. Mei F. (2008). Epac and PKA: a tale of two intracellular cAMP receptors. *Acta Biochim. Biophys. Sin. (Shanghai)* 40 651–662 18604457 10.1111/j.1745-7270.2008.00438.x PMC2630796

[B14] Cotlin L. F. Siddiqui M. A. Simpson F. Collawn J. F. (1999). Casein kinase II activity is required for transferrin receptor endocytosis. *J. Biol. Chem.* 274 30550–30556 10521437 10.1074/jbc.274.43.30550

[B15] Coxon F. P. Taylor A. (2008). Vesicular trafficking in osteoclasts. *Semin. Cell Dev. Biol.* 19 424–433 18768162 10.1016/j.semcdb.2008.08.004

[B16] Darios F. Connell E. Davletov B. (2007). Phospholipases and fatty acid signalling in exocytosis. *J. Physiol.* 585 699–704 17584839 10.1113/jphysiol.2007.136812 PMC2375517

[B17] Davies J. Warwick J. Totty N. Philp R. Helfrich M. Horton M. (1989). The osteoclast functional antigen, implicated in the regulation of bone resorption, is biochemically related to the vitronectin receptor. *J. Cell Biol.* 109 1817–1826 2477382 10.1083/jcb.109.4.1817 PMC2115816

[B18] Davletov B. Montecucco C. (2010). Lipid function at synapses. *Curr. Opin. Neurobiol.* 20 543–549 20655194 10.1016/j.conb.2010.06.008

[B19] DeSelm C. J. Miller B. C. Zou W. Beatty W. L. Van Meel E. Takahata Y. Klumperman J. Tooze S. A. Teitelbaum S. L. Virgin H. W. (2011). Autophagy proteins regulate the secretory component of osteoclastic bone resorption. *Dev. Cell* 21 966–974 22055344 10.1016/j.devcel.2011.08.016 PMC3244473

[B20] Donaldson J. G. (2009). Phospholipase D in endocytosis and endosomal recycling pathways. *Biochim. Biophys. Acta* 1791 845–849 19540357 10.1016/j.bbalip.2009.05.011 PMC2731818

[B21] Ganley I. G. Wong P. M. Gammoh N. Jiang X. (2011). Distinct autophagosomal-lysosomal fusion mechanism revealed by thapsigargin-induced autophagy arrest. *Mol. Cell* 42 731–743 21700220 10.1016/j.molcel.2011.04.024 PMC3124681

[B22] Gekle M. Mildenberger S. Freudinger R. Silbernagl S. (1995). Kinetics of receptor-mediated endocytosis of albumin in cells derived from the proximal tubule of the kidney (opossum kidney cells): influence of Ca^2+^ and cAMP. *Pflugers Arch.* 430 374–380 7491261 10.1007/BF00373912

[B23] Guilak F. Leddy H. A. Liedtke W. (2010). Transient receptor potential vanilloid 4: The sixth sense of the musculoskeletal system? *Ann. N. Y. Acad. Sci.* 1192 404–409 20392266 10.1111/j.1749-6632.2010.05389.x PMC3580043

[B24] Hansen S. H. Casanova J. E. (1994). Gs alpha stimulates transcytosis and apical secretion in MDCK cells through cAMP and protein kinase A. *J. Cell Biol.* 126 677–687 8045932 10.1083/jcb.126.3.677 PMC2120136

[B25] Hay J. C. (2007). Calcium: a fundamental regulator of intracellular membrane fusion? *EMBO Rep.* 8 236–240 17330068 10.1038/sj.embor.7400921 PMC1808041

[B26] Henriksen K. Bay-Jensen A. C. Christiansen C. Karsdal M. A. (2010). Oral salmon calcitonin – pharmacology in osteoporosis. *Expert Opin. Biol. Ther.* 10 1617–1629 20932224 10.1517/14712598.2010.526104

[B27] Horne W. C. Shyu J. F. Chakraborty M. Baron R. (1994). Signal transduction by calcitonin multiple ligands, receptors, and signaling pathways. *Trends Endocrinol. Metab.* 5 395–401 18407235 10.1016/1043-2760(95)92521-j

[B28] Hosoi N. Holt M. Sakaba T. (2009). Calcium dependence of exo- and endocytotic coupling at a glutamatergic synapse. *Neuron* 63 216–229 19640480 10.1016/j.neuron.2009.06.010

[B29] Inzerillo A. M. Zaidi M. Huang C. L. (2002). Calcitonin: the other thyroid hormone. *Thyroid* 12 791–798 12481944 10.1089/105072502760339352

[B30] Karsdal M. A. Martin T. J. Bollerslev J. Christiansen C. Henriksen K. (2007). Are nonresorbing osteoclasts sources of bone anabolic activity? *J. Bone Miner. Res.* 22 487–494 17227224 10.1359/jbmr.070109

[B31] Kopperud R. Krakstad C. Selheim F. Doskeland S. O. (2003). cAMP effector mechanisms. Novel twists for an “old” signaling system. *FEBS Lett.* 546 121–126 10.1016/s0014-5793(03)00563-5 12829247

[B32] Kruger M. C. Coetzee M. Haag M. Weiler H. (2010). Long-chain polyunsaturated fatty acids: selected mechanisms of action on bone. *Prog. Lipid Res.* 49 438–449 20600307 10.1016/j.plipres.2010.06.002

[B33] Lakkakorpi P. T. Lehenkari P. P. Rautiala T. J. Vaananen H. K. (1996). Different calcium sensitivity in osteoclasts on glass and on bone and maintenance of cytoskeletal structures on bone in the presence of high extracellular calcium. *J. Cell Physiol.* 168 668–677 8816921 10.1002/(SICI)1097-4652(199609)168:3<668::AID-JCP19>3.0.CO;2-V

[B34] Lee T. H. Linstedt A. D. (2000). Potential role for protein kinases in regulation of bidirectional endoplasmic reticulum-to-Golgi transport revealed by protein kinase inhibitor H89. *Mol. Biol. Cell* 11 2577–2590 10930455 10.1091/mbc.11.8.2577 PMC14941

[B35] Lochner A. Moolman J. A. (2006). The many faces of H89: a review. *Cardiovasc. Drug Rev.* 24 261–274 17214602 10.1111/j.1527-3466.2006.00261.x

[B36] Lucht U. (1973). Effects of calcitonin on osteoclasts *in vivo*. An ultrastructural and histochemical study. *Z. Zellforsch. Mikrosk. Anat.* 145 75–87 10.1007/BF00307190 4360462

[B37] Malgaroli A. Meldolesi J. Zallone A. Z. Teti A. (1989). Control of cytosolic free calcium in rat and chicken osteoclasts. The role of extracellular calcium and calcitonin. *J. Biol. Chem.* 264 14342–14347 2547794

[B38] Malhotra V. Campelo F. (2011). PKD regulates membrane fission to generate TGN to cell surface transport carriers. *Cold Spring Harb. Perspect. Biol.* 3 a005280. 10.1101/cshperspect.a005280 PMC3039530 21421913

[B39] Matsuo K. (2012). Osteocytes communicate with osteoclast lineage cells via RANKL. *IBMS BoneKEy* 9 39

[B40] Mellis D. J. Itzstein C. Helfrich M. H. Crockett J. C. (2011). The skeleton: a multi-functional complex organ: the role of key signalling pathways in osteoclast differentiation and in bone resorption. *J. Endocrinol.* 211 131–143 21903860 10.1530/JOE-11-0212

[B41] Mentaverri R. Kamel S. Brazier M. (2003). Involvement of capacitive calcium entry and calcium store refilling in osteoclastic survival and bone resorption process. *Cell Calcium* 34 169–175 12810059 10.1016/s0143-4160(03)00080-0

[B42] Mentaverri R. Yano S. Chattopadhyay N. Petit L. Kifor O. Kamel S. Terwilliger E. F. Brazier M. Brown E. M. (2006). The calcium sensing receptor is directly involved in both osteoclast differentiation and apoptosis. *FASEB J.* 20 2562–2564 17077282 10.1096/fj.06-6304fje

[B43] Morimoto R. Uehara S. Yatsushiro S. Juge N. Hua Z. Senoh S. Echigo N. Hayashi M. Mizoguchi T. Ninomiya T. Udagawa N. Omote H. Yamamoto A. Edwards R. H. Moriyama Y. (2006). Secretion of L-glutamate from osteoclasts through transcytosis. *EMBO J.* 25 4175–4186 16957773 10.1038/sj.emboj.7601317 PMC1570443

[B44] Murrills R. J. Dempster D. W. (1990). The effects of stimulators of intracellular cyclic AMP on rat and chick osteoclasts *in vitro*: validation of a simplified light microscope assay of bone resorption. *Bone* 11 333–344 1701319 10.1016/8756-3282(90)90089-h

[B45] Naro F. Perez M. Migliaccio S. Galson D. L. Orcel P. Teti A. Goldring S. R. (1998). Phospholipase D- and protein kinase C isoenzyme-dependent signal transduction pathways activated by the calcitonin receptor. *Endocrinology* 139 3241–3248 9645699 10.1210/endo.139.7.6112

[B46] Palacios N. Sanchez-Franco F. Fernandez M. Sanchez I. Villuendas G. Cacicedo L. (2007). Opposite effects of two PKA inhibitors on cAMP inhibition of IGF-I-induced oligodendrocyte development: a problem of unspecificity? *Brain Res.* 1178 1–11 17920050 10.1016/j.brainres.2007.07.018

[B47] Peters C. Mayer A. (1998). Ca2+/calmodulin signals the completion of docking and triggers a late step of vacuole fusion. *Nature* 396 575–580 9859992 10.1038/25133

[B48] Pizzo P. Lissandron V. Capitanio P. Pozzan T. (2011). Ca(2+) signalling in the Golgi apparatus. *Cell Calcium* 50 184–192 21316101 10.1016/j.ceca.2011.01.006

[B49] Risinger C. Bennett M. K. (1999). Differential phosphorylation of syntaxin and synaptosome-associated protein of 25 kDa (SNAP-25) isoforms. *J. Neurochem.* 72 614–624 9930733 10.1046/j.1471-4159.1999.0720614.x

[B50] Rosa J. M. Torregrosa-Hetland C. J. Colmena I. Gutierrez L. M. Garcia A. G. Gandia L. (2011). Calcium entry through slow-inactivating L-type calcium channels preferentially triggers endocytosis rather than exocytosis in bovine chromaffin cells. *Am. J. Physiol. Cell Physiol.* 301 C86–C98 21451100 10.1152/ajpcell.00440.2010

[B51] Shen D. Wang X. Xu H. (2011). Pairing phosphoinositides with calcium ions in endolysosomal dynamics: phosphoinositides control the direction and specificity of membrane trafficking by regulating the activity of calcium channels in the endolysosomes. *Bioessays* 33 448–457 21538413 10.1002/bies.201000152 PMC3107950

[B52] Shyu J. F. Shih C. Tseng C. Y. Lin C. H. Sun D. T. Liu H. T. Tsung H. C. Chen T. H. Lu R. B. (2007). Calcitonin induces podosome disassembly and detachment of osteoclasts by modulating Pyk2 and Src activities. *Bone* 40 1329–1342 17321230 10.1016/j.bone.2007.01.014

[B53] Smillie K. J. Cousin M. A. (2005). Dynamin I phosphorylation and the control of synaptic vesicle endocytosis. *Biochem. Soc. Symp.*, 72 87–97 15649133 10.1042/bss0720087 PMC2077358

[B54] Sossin W. S. Farah C. A. (2009). “Synaptic plasticity: diacylglycerol signalling role,” in *Encyclopedia of Neuroscience,* ed. Squire L. R. S.(Oxford: Academic Press) 747–755

[B55] Spilsberg B. Llorente A. Sandvig K. (2007). Polyunsaturated fatty acids regulate Shiga toxin transport. *Biochem. Biophys. Res. Commun.* 364 283–288 17942073 10.1016/j.bbrc.2007.09.126

[B56] Stenbeck G. Horton M. A. (2000). A new specialized cell-matrix interaction in actively resorbing osteoclasts. *J. Cell Sci.* 113 1577–1587 10751149 10.1242/jcs.113.9.1577

[B57] Stenbeck G. Horton M. A. (2004). Endocytic trafficking in actively resorbing osteoclasts. *J. Cell Sci.* 117 827–836 14762112 10.1242/jcs.00935

[B58] Stock J. L. Coderre J. A. (1984). Calcitonin enhances production of prostaglandins by stimulated human monocytes. *Prostaglandins* 27 771–779 6087420 10.1016/0090-6980(84)90014-5

[B59] Su Y. Chakraborty M. Nathanson M. H. Baron R. (1992). Differential effects of the 3’,5’-cyclic adenosine monophosphate and protein kinase C pathways on the response of isolated rat osteoclasts to calcitonin. *Endocrinology* 131 1497–1502 1324163 10.1210/endo.131.3.1324163

[B60] Teti A. (2011). Bone development: overview of bone cells and signaling. *Curr. Osteoporos. Rep.* 9 264–273 21948208 10.1007/s11914-011-0078-8

[B61] Tezuka K. Sato T. Kamioka H. Nijweide P. J. Tanaka K. Matsuo T. Ohta M. Kurihara N. Hakeda Y. Kumegawa M. (1992). Identification of osteopontin in isolated rabbit osteoclasts. *Biochem. Biophys. Res. Commun.* 186 911–917 1379809 10.1016/0006-291x(92)90832-6

[B62] Vaananen H. K. Laitala-Leinonen T. (2008). Osteoclast lineage and function. *Arch. Biochem. Biophys.* 473 132–138 18424258 10.1016/j.abb.2008.03.037

[B63] van der Eerden B. C. Hoenderop J. G. De Vries T. J. Schoenmaker T. Buurman C. J. Uitterlinden A. G. Pols H. A. Bindels R. J Van Leeuwen J. P. (2005). The epithelial Ca^2+^ channel TRPV5 is essential for proper osteoclastic bone resorption. *Proc. Natl. Acad. Sci. U.S.A.* 102 17507–17512 16291808 10.1073/pnas.0505789102 PMC1297662

[B64] Wojtal K. A. Hoekstra D Van Ijzendoorn S. C. (2008). cAMP-dependent protein kinase A and the dynamics of epithelial cell surface domains: moving membranes to keep in shape. *Bioessays* 30 146–155 18200529 10.1002/bies.20705

[B65] Xia S. L. Ferrier J. (1996). Localized calcium signaling in multinucleated osteoclasts. *J. Cell Physiol.* 167 148–155 8698832 10.1002/(SICI)1097-4652(199604)167:1<148::AID-JCP17>3.0.CO;2-7

[B66] Xiong J O’Brien C. A. (2012). Osteocyte RANKL: new insights into the control of bone remodeling. *J. Bone Miner. Res.* 27 499–505 22354849 10.1002/jbmr.1547 PMC3449092

[B67] Yamaki M. Nakamura H. Takahashi N. Udagawa N. Ozawa H. (2005). Transcytosis of calcium from bone by osteoclast-like cells evidenced by direct visualization of calcium in cells. *Arch. Biochem. Biophys.* 440 10–17 15993377 10.1016/j.abb.2005.05.021

[B68] Yamashita T. (2012). Ca(2+)-dependent regulation of synaptic vesicle endocytosis. *Neurosci. Res.* 73 1–7 22401840 10.1016/j.neures.2012.02.012

[B69] Yuan J. Akiyama M. Nakahama K. Sato T. Uematsu H. Morita I. (2010). The effects of polyunsaturated fatty acids and their metabolites on osteoclastogenesis *in vitro*. *Prostaglandins Other Lipid Mediat.* 92 85–90 20394833 10.1016/j.prostaglandins.2010.04.001

[B70] Zhang Z. Neff L. Bothwell A. L. Baron R. Horne W. C. (2002). Calcitonin induces dephosphorylation of Pyk2 and phosphorylation of focal adhesion kinase in osteoclasts. *Bone* 31 359–365 12231407 10.1016/s8756-3282(02)00834-7

[B71] Zhao H. Ito Y. Chappel J. Andrews N. W. Teitelbaum S. L. Ross F. P. (2008). Synaptotagmin VII regulates bone remodeling by modulating osteoclast and osteoblast secretion. *Dev. Cell* 14 914–925 18539119 10.1016/j.devcel.2008.03.022 PMC2480494

